# Intrahepatic Splenosis Mimicking Hepatocellular Carcinoma: A Case Series of Eleven Patients

**DOI:** 10.2174/0115734056443530260206213844

**Published:** 2026-04-21

**Authors:** Suzhen Li, Tianhao Zou, Zhiying Wang, Sisi Chen, Weimin Wang, Xing Zhou, Yang Gao, Guoliang Wang, Chen Zhang, Qichang Zheng, Shaobo Hu, Jianjun Xu

**Affiliations:** 1 Department of Gastroenterology, Wuhan Asia General Hospital, Wuhan 430056, China; 2 Liver Transplant Center, Union Hospital, Tongji Medical College, Huazhong University of Science and Technology, Wuhan 430022, China; 3 Department of Hepatobiliary Surgery, Union Hospital, Tongji Medical College, Huazhong University of Science and Technology, Wuhan 430022, China

**Keywords:** Intrahepatic splenosis, Splenic rupture, Hepatocellular carcinoma, Biopsy, Misdiagnosis, Multidisciplinary team

## Abstract

**Introduction:**

Intrahepatic splenosis is an extremely rare intrahepatic mass, which is easily misdiagnosed and mistreated. There are a few reports in the literature that intrahepatic splenosis mimicking hepatocellular carcinoma in a patient with elevated AFP.

This study aims to analyze the diagnosis and treatment strategies of intrahepatic splenosis.

**Materials and Methods:**

The clinical data of eleven patients with intrahepatic splenosis, diagnosed and treated at Wuhan Asia General Hospital and Union Hospital (Wuhan, China) between March 2012 and November 2024, were retrospectively analyzed. Enhanced CT imaging and enhanced MRI were used for the screening and diagnosis of liver lesions.

**Results:**

Of the eleven patients with intrahepatic splenosis, six cases were pure intrahepatic splenosis, and five cases included extrahepatic splenosis. Enhanced CT imaging or enhanced MRI showed intrahepatic splenosis lesions with uneven enhancement in the form of fast in and fast out. Two patients were misdiagnosed with hepatocellular carcinoma due to elevated AFP, but biopsy revealed intrahepatic splenosis, thus avoiding unnecessary resection. The size of the intrahepatic splenosis lesions ranged from 1.0 to 4.2 cm. None of the nine patients who underwent surgical resection had splenosis recurrences, and the patients with intrahepatic splenosis confirmed by liver biopsy did not show lesion progression during the active examination period.

**Discussion:**

Splenosis refers to the autotransplantation of viable splenic tissue into different anatomic compartments following splenic injury. The enhanced CT or MRI features of intrahepatic splenosis are similar to those of HCC. Selective hepatic arteriography may help differentiate intrahepatic splenosis from HCC. Percutaneous liver biopsy helps diagnose intrahepatic splenosis.

**Conclusion:**

In patients who have previously undergone splenectomy due to splenic trauma, it is important to consider the potential occurrence of intrahepatic splenosis upon the identification of intrahepatic lesions, and percutaneous liver biopsy is recommended. For individuals without clinical symptoms following a confirmed diagnosis of intrahepatic splenosis, no specific treatment is required.

## 1 INTRODUCTION

Splenosis refers to the autotransplantation of viable splenic tissue into different anatomic compartments following splenic injury. This condition typically arises after splenectomy, abdominal trauma, or splenic rupture [1]. Intra-abdominal splenosis most commonly involves the serosal surfaces of the small and large bowel, parietal peritoneum, and mesentery [2, 3]. However, intrahepatic splenosis is rare, with fewer than fifty cases reported in the literature to date [4-6]. Intrahepatic splenosis is a benign condition and does not warrant surveillance or intervention unless the patient is severely symptomatic. Diagnosing intrahepatic splenosis remains challenging, as patients are often asymptomatic or present with non-specific abdominal pain. Moreover, radiological findings can closely mimic other hepatic lesions, particularly hepatocellular carcinoma (HCC), adenoma, or focal nodular hyperplasia [7]. Furthermore, patients undergoing unnecessary operations may bear costs and morbidity. However, intrahepatic splenosis mimicking HCC in a patient with elevated AFP is seldom reported in the literature. This report describes a series of rare cases of intrahepatic splenosis mimicking HCC.

## 2 MATERIALS AND METHODS

### 2.1 Patients

Patients diagnosed with intrahepatic splenosis based on pathological findings were preliminarily included in the study. Patients without intrahepatic splenosis or splenic implantation without a pathological diagnosis were excluded. Ultimately, this retrospective analysis involved a total of eleven patients diagnosed with intrahepatic splenosis based on pathological findings. The treatment of these individuals took place from March 2012 to November 2024 at Wuhan Asia General Hospital and Union Hospital (Wuhan, China). Informed consent was secured from all participants involved in the study. This retrospective study was approved by the Ethical Review Committee of the Tongji Medical College (NO: IORG0003571). This study was conducted in full accordance with the ethical principles of the World Medical Association's Declaration of Helsinki. The study followed the Sex and Gender Equity in Re search guidelines (SAGER) to ensure appropriate inclusion and reporting of sex-related data.

### 2.2 Observation Indicators

The researchers extracted various data from the patients' medical records, including age, gender, symptoms, physical examination results, laboratory test outcomes, medical history, imaging characteristics, treatment protocols, pathological results, and follow-up information.

### 2.3 Imaging Acquisition and Analysis

Enhanced CT imaging and enhanced MRI were used for the screening and diagnosis of liver lesions. All MRI exams were performed on 3.0-T scanners; the extracellular contrast agent Gadobutrol was used for dynamic imaging; the hepatobiliary-phase imaging was performed using Gadoxetate disodium in 8/11 patients; standard parameters for T1/T2, DWI (b-values: 0, 50, 400, 800 s/mm^2^), and dynamic phases are listed. We explicitly state that all lesions were hypotintense on the hepatobiliary phase when this sequence was performed, a key differentiating feature from focal nodular hyperplasia.

### 2.4 Follow-up Protocol

Patients were assessed at the hospital three months post-surgery or biopsy, followed by evaluations every six months for the subsequent two years. After that period, they were monitored annually until the last follow-up in November 2024. A standard physical examination, blood tests, and imaging studies were conducted during each follow-up visit. At each follow-up visit, one of the imaging examinations, such as ultrasound, CT, or MRI, was used to assess the progress of splenosis. The final assessment categorized the patients based on whether splenosis had recurred.

## 3 RESULTS

### 3.1 Cohort Demographics and Clinical Features

Eleven patients diagnosed with intrahepatic splenosis were included in this retrospective study. Their basic clinical data were listed in Table **1**. The mean age of patients was 52 ± 9.2 years (range 41-76 years). All patients had a past medical history of splenectomy due to a traumatic rupture of the spleen. Three patients were infected with chronic viral hepatitis B. Two patients were infected with chronic viral hepatitis C. Presenting symptoms comprised abdominal pain and jaundice. Biochemical test documented an increase in alpha-fetoprotein (AFP), CA199, alanine aminotransferase, aspartate aminotrans-ferase, and γ-glutamyltransferase. Splenic implant lesions, ranging in number from 1 to 7, were found scattered in the liver, diaphragm, omentum, or peritoneum. Their diameter ranged from 1.0 to 4.2 cm. All patients underwent enhanced CT and MRI scans. Contrast-enhanced CT and MRI demons-trated rapid wash-in and wash-out enhancement. Diffusion-weighted imaging (DWI) demonstrated a high signal. At a specific stage of liver, the splenosis showed a low signal. Selective hepatic arteriography showed that the splenic implant lesions on the liver surface were not supplied by the hepatic artery.

Before the operation, patients No. 4 and No. 5 received an ultrasound-guided liver biopsy of the liver lesion, which showed a blood sinusoid structure and focal aggregation of lymphocytes, and failed to confirm the diagnosis of intrahepatic splenosis. Patients No. 1 to No. 9 underwent surgical treatment to remove the lesions, and postoperative pathology confirmed the diagnosis of splenosis. Patients No. 10 and No. 11 underwent liver biopsy, and pathology confirmed the diagnosis of intrahepatic splenosis. Patient No. 10 was diagnosed with hepatitis B and received entecavir treatment. During the three-month follow-up, alpha-fetoprotein decreased from 49.1 μg/L to 3.6 μg/L. Patient No. 11 was diagnosed with hepatitis C and received sofosbuvir and velpatasvir tablets treatment. During the three-month follow-up, alpha-fetoprotein decreased from 29.9 μg/L to 2.8 μg/L. Consequently, both patients were spared unnecessary resection and were managed conservatively with follow-up.

### 3.2 Index Case: Intrahepatic Splenosis with Elevated AFP

**Fig. (1):**
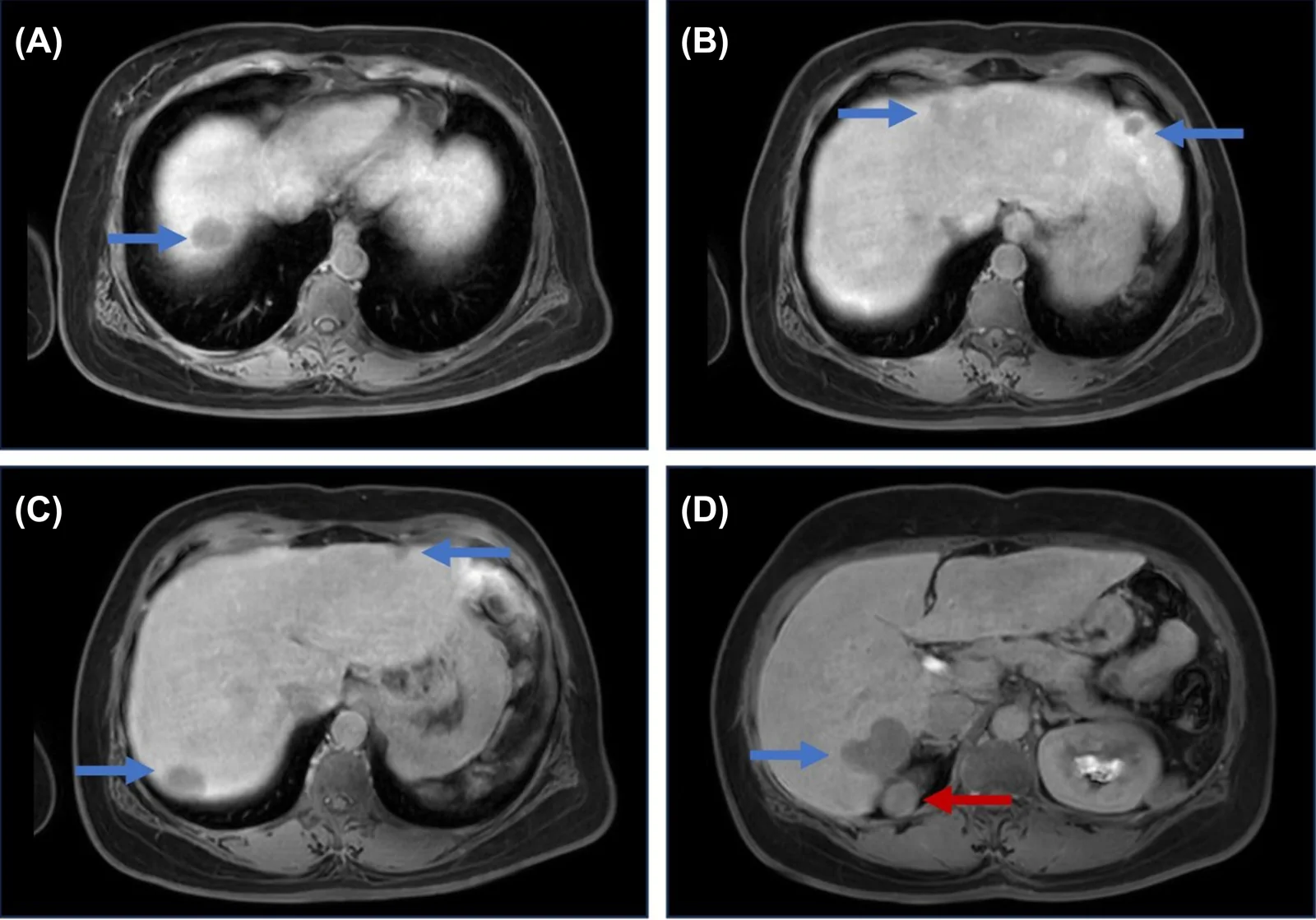
Splenic implant lesions distribution in MRI scans. (**A**) Intrahepatic splenosis lesion (blue arrow) No.1, (**B**) Intrahepatic splenosis lesions No.2 and No.3, (**C**) Intrahepatic splenosis lesions No.4 and No.5, (**D**) Intrahepatic splenosis lesions No.6 and visceral peritoneum splenic implant lesion (red arrow).

**Fig. (2):**
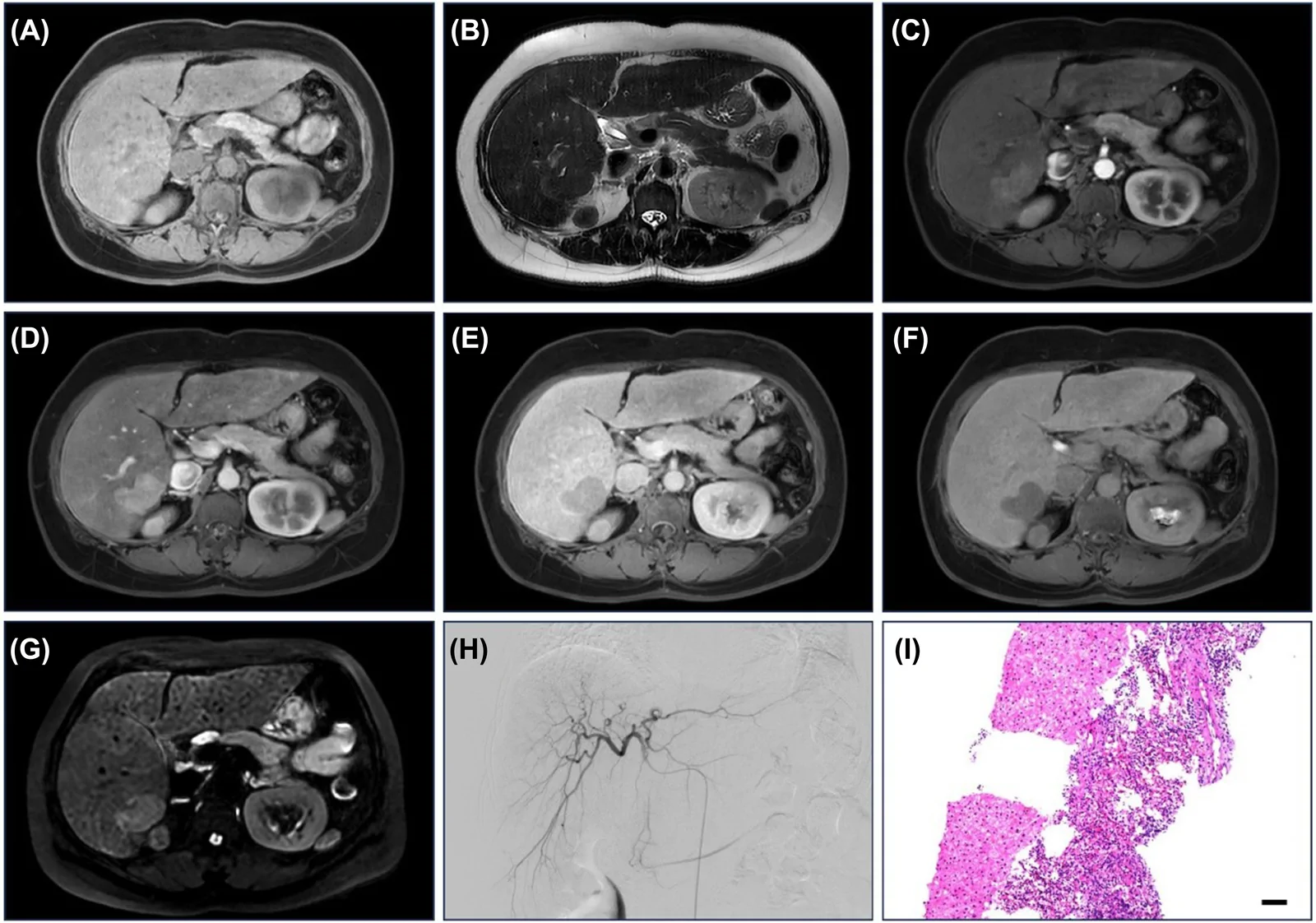
Clinical data of a typical splenosis case. (**A**) T1 weighted plain scan, (**B**) T2 weighted plain scan, (**C**) Arterial stage, (**D**) Portal vein stage, (**E**) Delayed stage, (**F**) Liver specific stage, (**G**) Diffusion-weighted imaging, (**H**) Selective hepatic arteriography, (**I**) Histopathology showing hepatic tissue separated by a fibrous capsula from the highly vascularized lymphoid tissue with follicular areas. H&E, ×40. Scale bar: 50 μm.

A 48-year-old female patient (Patient No. 11) was hospitalized following the identification of multiple lesions in the liver and visceral peritoneum one and a half months earlier (Fig. **1**). The patient had a history of cholecystectomy, which was performed to treat gallstones. In addition, the patient had a prior history of traumatic splenic rupture, which was treated with a splenectomy and blood transfusion. The patient tested positive for anti-hepatitis C antibodies and elevated levels of hepatitis C RNA. Therefore, the patient received antiviral therapy with sofosbuvir and velpatasvir tablets. MRI showed that the lesions were distributed across the liver surface and visceral peritoneum (Fig. **1**), with slightly longer circular T1 (Fig. **2A**) and slightly longer T2 (Fig. **2B**) signals. The larger one was located in the S6 segment, with a maximum cross-section of about 42×28 mm. Enhanced MRI showed uneven enhancement in the form of rapid wash-in and wash-out enhancement. (Fig. **2C**-**E**). At a specific stage of liver, the lesions showed low signal (Fig. **2F**). DWI demonstrated a high signal (Fig. **2G**). The intrahepatic blood vessels were normal, and the intrahepatic bile ducts were not dilated. There was a slight accumulation of fluid around the liver. The gallbladder and spleen were not shown. The imaging diagnosis considered that the intrahepatic lesion might be HCC. Laboratory tests showed abnormally elevated levels of AFP (29.9 μg/L), CA199 (38.8 U/ml), alanine aminotransferase (52 U/L), aspartate aminotransferase (44 U/L), and γ-glutamyltransferase (467 U/L). The level of albumin (32.1 g/L) was decreased. The blood clotting function test showed prothrombin time (14.8 s) was prolonged. No abnormalities were found in routine blood tests. The liver function reserve test showed that the retention rate of indocyanine green was 40.1% in 15 minutes.

Based on the typical imaging findings, elevated AFP and hepatitis C virus infection as described in the guidelines for the diagnosis and treatment of primary liver cancer, the patient was initially diagnosed with HCC. Subsequently, the patient underwent a multidisciplinary team consultation. It was considered that the lesions in this patient were primarily on the surface of the liver and the visceral peritoneum, and that the patient had undergone splenectomy. Lesions on the liver surface and visceral peritoneum did not exclude the possibility of splenosis. In addition, the patient's elevated AFP levels might be caused by active hepatitis. The patient was recommended for a liver biopsy. Selective hepatic arteriography (Fig. **2H**) and transcatheter arterial embolization were performed at the same time as puncture biopsy, considering that splenic implantation on the liver surface may not be supplied by the hepatic artery, and to reduce bleeding due to puncture. The pathological diagnosis of biopsy was considered to be intrahepatic splenosis (Fig. **2I**). Therefore, the patient fortunately avoided unnecessary surgery and was recommended for follow-up.

After three months of antiviral therapy with sofosbuvir and velpatasvir tablets and follow-up, the patient's AFP and aminotransferase levels were reduced to normal. At the same time, there was no significant progress in intrahepatic splenosis.

### 3.3 Pathological Findings

Pathological evaluation confirmed intrahepatic splenosis in all eleven patients. The hepatic lesions ranged from 1.0 to 4.2 cm in size and shared uniform pathological features. Grossly, the lesions were well-circumscribed, soft, and friable, with dark red to grayish-brown cut surfaces. Microscopic examination revealed well-defined red and white pulp. The white pulp contained lymphoid follicles, while the red pulp showed irregular splenic cords and a network of irregularly shaped vascular sinuses.

### 3.4 Treatment Outcomes and Follow-up

None of the patients developed serious complications or died during the perioperative period. As of November 2024, the median duration of the follow-up was 24 months (range: 3-60 months). None of the nine patients who underwent surgical resection had splenosis recurrences. Patients No. 10 and No. 11 with puncture biopsy showed no significant progression of splenosis lesions during three months of follow-up.

## 4 DISCUSSION

Splenosis arises through several mechanisms, with direct implantation following splenic trauma being the most common pathway in our cohort, consistent with existing literature [4]. Firstly, direct dissemination and implantation of splenic tissue fragments following splenic injury. Secondly, during abdominal cavity irrigation with substantial amounts of normal saline, small splenic tissue fragments may migrate to areas distant from the spleen and become implanted. Thirdly, myeloid cells from the spleen can be transported to the liver via the splenic vein and portal vein, a method of splenosis in the liver that is relatively uncommon in clinical practice. In the study involving seven patients, the majority of splenosis were found on the liver surface, with only one focus in the central hepatic parenchyma. Thus, it was concluded that most splenosis in the liver was likely fragments of splenic tissue directly planted on the hepatic serous membrane, which then became compressed and integrated into the liver tissue due to the pressure from the abdominal wall, diaphragm, or adjacent tissues during growth. Given the spleen's significant role in immune function, splenectomy often leads to severe complications such as explosive infections. To preserve some of the spleen's immune function and minimize the risk of such infections, autogenous splenic tissue fragments can be implanted into the omentum during surgery. Based on this theoretical basis, it could be seen that splenosis has certain clinical significance.

The distribution of splenosis lesions is closely related to the anatomical context of the initial injury, which explains the predominance of intra-abdominal locations in our patients. After a splenectomy, approximately 90% of splenosis lesions are found within the abdominal cavity, predominantly affecting the serosal membrane of the small intestine, omentum, parietal peritoneum, mesentery, and pelvic cavity [8]. Given that the spleen is anatomically situated in the left upper abdomen, it is more common for spleen tissue to spread and implant in the left middle and upper abdominal regions. This is less frequently observed in the liver, diaphragm, or other distant organs [9]. In cases where spleen injury is accompanied by thoraco-abdominal trauma, splenosis lesions can appear in the chest [10].

Clinically, intrahepatic splenosis is often asymptomatic, as observed in most of our cases, but its presentation can vary depending on lesion location and size. Intrahepatic or intra-abdominal splenosis typically presents no distinctive clinical signs and is often discovered incidentally during routine examinations [11]. Most patients remain asymptomatic, though some may exhibit symptoms such as abdominal mass, pain, distension, or intestinal obstruction, depending on the splenosis location or size [12]. Additionally, if spleen tissue implants in the chest or lung, symptoms like chest pain, hemoptysis, tightness, and cough may occur [10].

Accurate diagnosis relies heavily on multimodality imaging, and our cases underscore the pivotal role of contrast-enhanced CT/MRI alongside selective angiography in differentiating splenosis from HCC. The diagnostic imaging techniques for splenosis in the liver primarily encompass B ultrasound, CT scans, MRI scans, and 99mTc-DRBC imaging [13, 14]. Additionally, percutaneous liver biopsy can be considered if a conclusive diagnosis is needed. Understanding the imaging features of splenosis in the liver is crucial for accurate clinical diagnosis. On B ultrasound, splenosis lesions in the liver often appear as round or oval, low-echo nodules with clear margins, uniform echogenicity, and significant blood flow signals. CT imaging reveals uniform nodules or masses within the hepatic tissue, typically round or oval with distinct borders, and CT values slightly lower than those of normal liver tissue, ranging from 40 to 70 HU [15, 16]. Enhancement patterns at the arterial phase vary with lesion size—nodules ≤3 cm generally show uniform enhancement, whereas those >3 cm often exhibit uneven or mottled enhancement due to variable blood flow velocity and irregular arrangement of red and white pulp [17]. In the portal phase, these lesions demonstrate consistent enhancement with a slight reduction in the retarder. MRI results revealed a consistent lesion signal on the MRI plain scan, displaying a low signal on T1-weighted images, a high signal on T2-weighted images, and a high signal on DWI and a low signal on the corresponding ADC maps, resembling the liver's signal pattern. The contrast-enhanced MRI findings were comparable to those observed in the enhanced CT scan. The 99mTc-DRBC imaging technique leverages the ability of monocyte macrophages in the spleen's red pulp to selectively ingest and eliminate abnormal red blood cells, allowing for spleen tissue visualization [18]. Consequently, some researchers proposed that 99mTc-DRBC imaging could serve as the “gold standard” for non-invasive ectopic splenosis diagnosis, due to the spleen's radioactive concentration being 2 to 4 times greater than that of the liver [19]. This specificity makes 99mTc-DRBC imaging particularly useful for identifying intrahepatic splenosis. In this report, selective hepatic angiography confirmed that the angiographic feature of intrahepatic splenic implantation is the presence of parasitic arterial supply from the phrenic artery or intercostal artery to the lesion around the liver, without proper hepatic artery supply.

The key clinical challenge lies in distinguishing intrahepatic splenosis from malignant hepatic tumors, particularly in patients with concurrent hepatitis or elevated AFP, as highlighted by our diagnostic experience. By integrating the history of splenectomy or spleen removal with the imaging features from various diagnostic tests, a clinical diagnosis of intrahepatic splenosis can be established. However, distinguishing it from both benign and malignant liver tumors in clinical settings is crucial, particularly for patients with a history of hepatitis, as there is a tendency for it to be misidentified as HCC.

Management of intrahepatic splenosis should be conservative in asymptomatic patients, as evidenced by the favorable outcomes in our biopsy-managed cases. A definitive diagnosis of intrahepatic splenosis is essential for determining appropriate follow-up treatment. The splenic tissue located in an ectopic site can often function similarly to a normal spleen, partially compensating for its roles. Given that splenosis lesions typically progress slowly, removal of intrahepatic splenosis is generally advised only if clinical symptoms are present. Surgical intervention is warranted when symptoms such as acute abdominal pain due to hemorrhage or infarction, persistent epigastric discomfort, or a sizable splenosis lesion impairing liver function occur [20, 21].

This study, as a retrospective analysis, has several inherent limitations. First, the sample size was small, which reflects the rarity of intrahepatic splenosis. Second, the retrospective design carries inherent risks of bias due to its dependence on historical medical records, potentially resulting in incomplete data or information gaps. Future research should focus on the following directions. Multi-center collaborations with larger sample sizes are needed to better characterize the natural history, imaging features, and long-term progression of intrahepatic splenosis. Additionally, efforts to develop optimized, non-invasive diagnostic workflows and differential diagnostic models, potentially incorporating AI-assisted imaging, could improve accuracy and reduce reliance on invasive procedures. Long-term, systematic prospective follow-up of confirmed cases would also help clarify lesion growth rates and their impact on liver function and adjacent structures, thereby informing evidence-based clinical management.

## CONCLUSION

The enhanced CT or MRI features of intrahepatic splenosis are similar to those of HCC. Selective hepatic arteriography may help differentiate intrahepatic splenosis from HCC. Percutaneous liver biopsy helps diagnose intrahepatic splenosis. For individuals without clinical symptoms following a confirmed diagnosis of intrahepatic splenosis, no specific treatment is required. This serial case report enhances clinicians' grasp of intrahepatic splenosis, including its concept, diagnostic methods, and treatment strategies, offering valuable insights for future cases.

## Figures and Tables

**Table 1:** Clinical data of intrahepatic splenosis patients.

**S.No.**	**Age**	**Gender**	**Symptom**	**MRI/CT Image Feature**	**Maximal Diameter**	**Primary Diagnosis**	**Treatment**
1	56y	Male	Upper abdominal pain	Multiple liver surface lesions, with the largest one in the S6 segment	38mm	HCC	Hepatectomy
2	76y	Male	No	Multiple liver surface lesions, with the largest one in the S3 segment	25mm	HCC	LH
3	41y	Female	No	Multiple liver surface lesions, with the largest one in the S2 segment	23mm	Angioleiomyolipoma or HCC?	LH
4	52y	Female	Right upper abdominal pain	Multiple liver surface and diaphragm lesions, with the largest one in the S2 segment	38mm	HCC with diaphragmatic metastasis	Hepatectomy and resection of the diaphragm lesion
5	50y	Female	No	Multiple liver surface lesions, with the largest one in the S3 segment	35mm	HCC	LH
6	49y	Male	Jaundice	Multiple liver surface and diaphragm lesions, with the largest one in the S3 segment	41mm	HCC with diaphragm metastasis	Hepatectomy and resection of the diaphragm lesion
7	45y	Female	No	Multiple liver surface lesions, with the largest one in the S6 segment	20mm	HCC	LH
8	59y	Male	No	One liver surface lesion in the S3 segment	26mm	HCC	LH
9	49y	Male	Right upper abdominal pain	Multiple liver surface and omentum lesions, with the largest one in the S6 segment	38mm	HCC with omentum metastasis	LH and resection of the omental lesion
10	52y	Male	No	Multiple liver surface and omentum lesions, with the largest one in the S2 segment	25mm	HCC with omentum metastasis	Liver biopsy
11	48y	Female	No	Multiple liver surface and visceral peritoneum lesions with the largest one in the S6 segment	42mm	HCC	Liver biopsy and transcatheter arterial embolization

**Abbreviations:** CT: computed tomography; HCC: hepatocellular carcinoma; LH: laparoscopic hepatectomy; MRI: magnetic resonance imaging.

## Data Availability

The data and supportive information are available within the article.

## LIST OF ABBREVIATIONS

HCC: Hepatocellular Carcinoma
DWI: Diffusion-weighted Imaging

## STANDARDS OF REPORTING

STROBE Guidelines were followed.

## References

[1] Toh W.S., Chan K.S., Lyn Ding C.S., Tan C.H., Shelat V.G. (2020). Intrahepatic splenosis: A world review.. Clin. Exp. Hepatol..

[2] Guarneri A., Rufini V., Schinzari G., Rindi G., Leccisotti L. (2024). Atypical presentation of splenic tissue mimicking neuroendocrine tumor relapse.. Clin. Nucl. Med..

[3] Marrana F., Tavares C., Furtado A., Moreira Marques T., Faria G. (2025). From a suspicious liver mass to splenosis: A case report.. Cureus.

[4] Luo X., Zeng J., Wang Y., Min Y., Shen A., Zhang Y., Deng H., Gong N. (2019). Hepatic splenosis: Rare yet important – A case report and literature review.. J. Int. Med. Res..

[5] Umeda I., Tomihara H., Maeda S., Kitagawa A., Miyamoto K., Kawabata R., Nishikawa K., Noura S., Yasuhara Y., Miyamoto A. (2023). A rare intrahepatic splenosis mimicking hepatocellular carcinoma: A case report.. Mol. Clin. Oncol..

[6] Pessarelli T., Crespi S., Antonelli B., Maggioni M., Iavarone M. (2022). Hepatic splenosis mimicking hepatocellular carcinoma in metabolic associated fatty liver disease.. Dig. Liver Dis..

[7] Guo S., Liu J., Zheng S. (2022). Intrahepatic splenosis mimicking hepatocellular carcinoma.. Clin. Gastroenterol. Hepatol..

[8] Degheili J.A., Abou Heidar N.F. (2019). Pelvic splenosis—A rare cause of pelvic mass.. Clin. Case Rep..

[9] Seguchi S., Yue F., Asanuma K., Sasaki K. (2015). Experimental splenosis in the liver and lung spread through the vasculature.. Cell Tissue Res..

[10] Mahmoudian B., Javadrashid R., Fakhrjoo A. (2015). Intrathoracic splenosis: A rare false-positive cause of somatostatin imaging in characterization of solitary pulmonary nodule.. Clin. Nucl. Med..

[11] Gandhi D., Sharma P., Garg G., Songmen S., Solanki S., Singh T. (2020). Intrahepatic splenosis demonstrated by diffusion weighted MRI with histologic confirmation.. Radiol. Case Rep..

[12] Tulinský L., Ihnát P., Mitták M., Guňková P., Zonča P. (2016). Intrathoracic splenosis – Lesson learned: A case report.. J. Cardiothorac. Surg..

[13] Reisner D.C., Burgan C.M. (2018). Wandering spleen: An overview.. Curr. Probl. Diagn. Radiol..

[14] Lau S.Y., Lao W.T. (2025). Imaging findings of a rare intrahepatic splenosis, mimicking hepatic tumor.. Diagnostics.

[15] Li T., Yang X.Y., Tang Z.Y. (2015). Intrahepatic and intraperitoneal splenosis mimicking hepatocellular carcinoma with abdominal wall metastasis in a patient with hepatitis C cirrhotic liver.. Surgery.

[16] Zhang H.H., Tan X.Z. (2024). Dual-energy CT of intrahepatic splenosis.. AJR Am. J. Roentgenol..

[17] Verma R., Mupparaju V., Nair S.P. (2023). Intrahepatic splenosis: Benign but can be misdiagnosed.. Clin. Gastroenterol. Hepatol..

[18] Bilello J., Kulak O., Selvarajan S., Tholey D., Civan J. (2023). A rare red herring: Intrahepatic splenosis masquerading as hepatocellular carcinoma.. ACG Case Rep. J..

[19] Tsai L.L., Kaliannan K., Mortele K.J. (2015). Gallbladder splenosis: A hereto unreported mimicker of a gallbladder neoplasm.. Clin. Imaging.

[20] Guo L., Shen G. (2024). Gastric fundus splenosis mimicking malignancy on FDG PET/CT.. Clin. Nucl. Med..

[21] Pugliesi R.A., Vernuccio F., Maino C., Matteini F., Blandino A.A., Brancatelli G., Cannella R. (2025). Lesions located at the liver periphery: A stepwise cross-sectional imaging approach toward diagnosis.. Eur. J. Radiol..

